# Supplementary material for: The curve not taken: Effects of COVID-19 international comparison news
Source: PLoS One. 2022 Aug 11;17(8):e0271041. doi: 10.1371/journal.pone.0271041 (PMC9371605; doi:10.1371/journal.pone.0271041)
Supplement: S1 Table — (PDF) [file pone.0271041.s001.pdf]

S1 Table. ANOVA results summary.

	Knowledge of policy differences (S.K. vs. U.S.)			Attitudes about U.S. response		
	Testing & Tracing	Lockdown misperception	Policy similarity misperception	Policy support	Presidential Blame	Trust in health experts
Treatment (df = 2)	***	***	*	**	**	*
Political party (df = 2)	**	***	***	***	***	**
Treatment x Political party (df = 4)	*	***				
Intercept (df = 1)	***	***	***	***	***	***

Note. The three non-hypothesized experimental factors (not shown here) were also included in all models for control purposes as main effects and two-way interactions with the treatment. * $p < .05$, ** $p < .01$, *** $p < .001$, one-tailed.
