# Supplementary material for: The curve not taken: Effects of COVID-19 international comparison news
Source: PLoS One. 2022 Aug 11;17(8):e0271041. doi: 10.1371/journal.pone.0271041 (PMC9371605; doi:10.1371/journal.pone.0271041)
Supplement: Attachment — Submitted filename: Response to Reviewers.docx [file pone.0271041.s002.docx]

Response to Reviewers

May 5, 2021

Seonwoo (Sun) Kim, Ph.D. student

Manship School of Mass Communication

Baton Rouge, LA 70803

**Dear Dr. Nanda,**

**We are delighted to be invited to revise our manuscript in light of the reviews. We appreciate the close reading of our manuscript and constructive feedback. We believe we have addressed all points raised and we believe these edits leave our manuscript much improved. Below we walk through the changes we made.**

1. Please ensure that your manuscript meets PLOS ONE's style requirements, including those for file naming. The PLOS ONE style templates can be found at

https://journals.plos.org/plosone/s/file?id=wjVg/PLOSOne_formatting_sample_main_body.pdf and

https://journals.plos.org/plosone/s/file?id=ba62/PLOSOne_formatting_sample_title_authors_affiliations.pdf

2. Please ensure that you refer to Figure 1 in your text as, if accepted, production will need this reference to link the reader to the figure.

**We added the reference to Figure 1 in the first paragraph of the “Results” section as follows:**

**"The RQ test used the interactions between party preference and the treatment in each model (see Fig 1)."**

3.We note that Supporting Information S1, S2 and S3 in your submission contain copyrighted images.

**We removed the copyrighted images from all stimuli and replaced them with equivalent images that we created.**

Queries:

1) Is it possible to always address the international news and policy news simultaneously?

**This is a good point and we believe that more theorizing and empirical work needs to be done to better distinguish between international news and policy news coverage. Even though the combination of international news and policy news is a promising form of news content, the media cover each news section separately (i.e., international news section & political news). Researchers have also studied them as independent research subjects. Thus, many potential effects remain to be seen. However, this study experimentally found strong evidence to support the benefits of international comparison news. In addition, depending on characteristics of issue and comparison characteristics, international policy comparison news effect would not work. We added it to discussion section further with highlighted marks.**

2) How far this article will help in improving policy?

**We thank the reviewer for raising this point. In our manuscript, we argue that international comparison news coverage may increase democratic accountability by providing comparisons that shed light on how other countries address specific issues, such as the Covid-19 crisis. International comparison news coverage may also help political elites and institutions choose desirable policies while warning citizens against malpractice, injustice, and misdeeds. Informed** **citizenry, accurate attribution of policy evaluation to politicians, and trust in experts are all expected to improve the quality of domestic policies, potentially contributing to a healthy broadening of range of policy options under debate. We added the example of gun control policy in the discussion section to show how comparing domestic policy to the policies of other countries may prove advantageous for a variety of reasons.**

3) Is the three-level treatment factor (international policy comparison news: policy comparison, outcome comparison, and a control condition with no international comparison) are significant to each other?

**We report the post-hoc analyses in the three-level treatment factor in the result section for each outcome. More importantly, the key take-away for journalists from this three level factor is that our results suggest international comparison news is more beneficial if it compares policies, not just outcomes. We added some discussion of these implications in the discussion section.**

4) How these samples play an vital role in external validity?

**As with any experiment using a convenience sample, external validity is a significant limitation. We have added further acknowledgment of this limitation in the methods and results sections, while noting that MTurk convenience samples do have the advantage of greater demographic diversity compared to undergraduate convenience samples.**

Sincerely,

Seonwoo (Sun) Kim
