# Supplementary material for: The curve not taken: Effects of COVID-19 international comparison news
Source: PLoS One. 2022 Aug 11;17(8):e0271041. doi: 10.1371/journal.pone.0271041 (PMC9371605; doi:10.1371/journal.pone.0271041)
Supplement: Attachment — Submitted filename: Response to Reviewers.docx [file pone.0271041.s004.docx]

**Response to Reviewers**

The curve not taken

Due July 14, 2022

Highlight edited text in yellow

We thank you for inviting a third round of minor revisions on this manuscript. We have reported a) how sample size was determined and b) the results of the sensitivity analysis to demonstrate the size of effect this study's sample was powered to detect. Please see below to respond to the comments point by point.

**Editor 1. How please report in text how sample size was determined**

⟶ As we responded in the second revision, we reported how we decided our sample size in the method section as follows, "The sample size decision was based on available resources and a rule of thumb for media effects experiments of 50 participants per cell [1]."

**Editor 2. Report in text the results of the sensitivity analysis to demonstrate the size of effect your sample was powered to detect.**

⟶ Thank you for pointing out the missing information in the text. We added the power analysis result in the method section as follows, "Retrospectively, we ran a power analysis based on an effect size of Cohen’s f = .14 from a solutions journalism experiment [2] and .95 power using G*power application, resulting in a required sample size of 792. The retrospective power analysis confirmed that our sample size is appropriate."

**Journal Requirements. Reference list check.**

⟶ We carefully reviewed our references again, and found no citation of any retracted paper in this manuscript. We've added one citation [1] in this revision round and yellow-highlighted it in the manuscript. Also, we used Preflight Analysis and Conversion Engine (PACE) digital diagnostic tool to check whether the fig1 meets PLOS requirements and optimize it through the service. We have replaced the previous figure file with the image updated through PACE.

**References**

1.	VanVoorhis CW, Morgan BL. Understanding power and rules of thumb for determining sample sizes. Tutorials in Quantitative Methods for Psychology. 2007;3(2):43-50. doi: 10.20982/tqmp.03.2.p043.

2.	McIntyre K. Solutions journalism: The effects of including solution information in news stories about social problems. Journalism Practice. 2019;13(8):1029-33. doi: 10.1080/17512786.2019.1640632.
